# Whole-Tissue Deconvolution and scRNAseq Analysis Identify Altered Endometrial Cellular Compositions and Functionality Associated With Endometriosis

**DOI:** 10.3389/fimmu.2021.788315

**Published:** 2022-01-05

**Authors:** Daniel G. Bunis, Wanxin Wang, Júlia Vallvé-Juanico, Sahar Houshdaran, Sushmita Sen, Isam Ben Soltane, Idit Kosti, Kim Chi Vo, Juan C. Irwin, Linda C. Giudice, Marina Sirota

**Affiliations:** ^1^ Bakar Computational Health Sciences Institute, University of California, San Francisco, San Francisco, CA, United States; ^2^ Center for Reproductive Sciences, University of California, San Francisco, San Francisco, CA, United States; ^3^ Department of Pediatrics, Division of Neonatology, University of California, San Francisco, San Francisco, CA, United States

**Keywords:** endometriosis, deconvolution, bulk tissue transcriptomics, single-cell analysis, eutopic endometrium

## Abstract

The uterine lining (endometrium) exhibits a pro-inflammatory phenotype in women with endometriosis, resulting in pain, infertility, and poor pregnancy outcomes. The full complement of cell types contributing to this phenotype has yet to be identified, as most studies have focused on bulk tissue or select cell populations. Herein, through integrating whole-tissue deconvolution and single-cell RNAseq, we comprehensively characterized immune and nonimmune cell types in the endometrium of women with or without disease and their dynamic changes across the menstrual cycle. We designed metrics to evaluate specificity of deconvolution signatures that resulted in single-cell identification of 13 novel signatures for immune cell subtypes in healthy endometrium. Guided by statistical metrics, we identified contributions of endometrial epithelial, endothelial, plasmacytoid dendritic cells, classical dendritic cells, monocytes, macrophages, and granulocytes to the endometrial pro-inflammatory phenotype, underscoring roles for nonimmune as well as immune cells to the dysfunctionality of this tissue.

## 1 Introduction

Human endometrium is a complex tissue that remodels during the menstrual cycle under the regulation of ovarian-derived steroid hormones. It is characterized by phenotypic changes in diverse cell groups and changes in their relative abundance by cell proliferation and infiltration (1). Endometriosis is a common, steroid-hormone-dependent disorder in which endometrial-like tissue invades pelvic organs, eliciting an inflammatory response and fibrosis, resulting in chronic pelvic pain and/or infertility. The latter is due mainly to abnormal eutopic endometrium (within the uterus) that is inhospitable to embryo implantation (2).

Previous bulk RNAseq and microarray analyses revealed altered transcriptomic profiles in the eutopic endometrium of women with versus without endometriosis (3–5). With disease, the eutopic endometrium displays a pro-inflammatory transcriptomic feature and fails to elicit normal steroid hormone responses that are essential for endometrial transformation (4, 6–8). This pro-inflammatory feature was also observed in the microarray and RNAseq profiles of isolated endometrial stromal fibroblasts (eSFs), mesenchymal stem cells (eMSCs), and macrophages (9, 10). However, the full complement and abundance of cell types contributing to the pro-inflammatory feature have yet to be identified and are addressed herein.

While single-cell (sc)RNAseq characterization can provide insights into the phenotypes of endometrial cell populations, current costs of this technology can be prohibitive for profiling samples at the scale required for the context of endometrial disorders. Accurate profiling in this context requires sufficient sampling in both disease and control individuals across the diverse hormonal milieu of the menstrual cycle. Even though evolving multiplexing strategies can help mitigate the high cost of single-cell technologies, deconvoluting whole tissue level data into cell types provides a promising alternative (11–20), where insights such as abundance variation can be derived with high statistical power from previously characterized bulk tissue samples, particularly those involving clinical samples for which prospective large-scale collection and phenotyping would require major investments of time and effort. Cell-type deconvolution relies on using appropriate cell-type signatures for the tissue of interest. While one strategy is to apply tissue-specific signatures derived from sorted cells or scRNAseq (21–25), it is limited by cell types known to the tissue, availability of signatures, and batch effects from different technologies used to derive the signatures (15), and often does not allow for discovery of new cell types.

To leverage the advantages and overcome the limitations of these approaches, in the current study, we used both whole tissue deconvolution analysis (26) and scRNAseq analyses to characterize the human endometrium from women with or without endometriosis. A signature compendium of 64 classical human cell types derived from diverse organs in 6 human tissue consortia were used, and a gene set enrichment-based deconvolution method was adapted (26). The applicability of each signature to the human endometrium was evaluated by building statistical metrics using scRNAseq endometrial data obtained from women without endometriosis (27). In addition to guiding data interpretation, signature evaluation prompted in-depth single cell level identification and annotation of 13 immune cell type/subtypes in healthy endometrium, including those whose identities and functions have been less well characterized and explored in endometrial biology. Herein, we present a comprehensive characterization of the cellular composition of the human endometrium across the menstrual cycle in women with and without endometriosis and the identification of cell types with altered abundance in one or multiple menstrual cycle phases of women with disease.

## 2 Results

### 2.1 Traditional Differential Expression Analysis Identifies Immune Pathways Associated With Endometriosis Across the Menstrual Cycle

**Figure 1:**
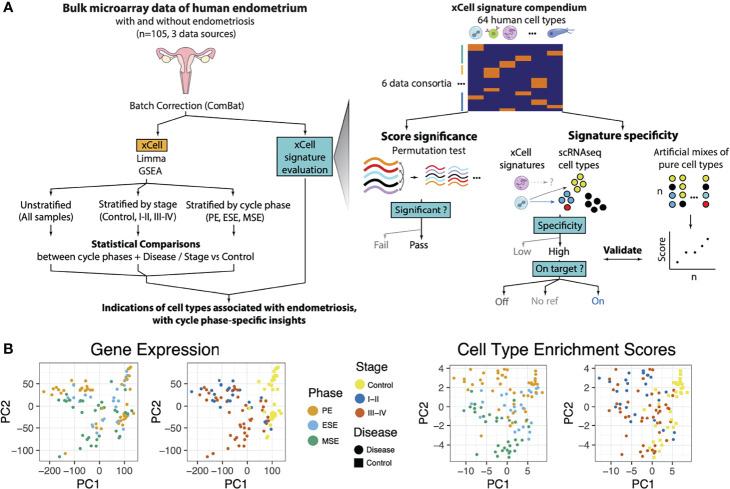
Analysis and Data Overview. **(A)** Experimental overview showing how endometrial tissue transcriptome microarray data were processed and analyzed. Data were normalized (see below) and batch corrected. Then, differential gene expression (DGE) analysis was performed with log-fold change outputs run through pathway enrichment analysis. Similarly, cell-type deconvolution of these bulk tissue samples was performed and validated and then analyzed for differential enrichments between sample groups. Each of these analyses was run for various stratifications (subsets) of the samples and targeting differences between distinct groupings for each stratification. These analyses were used to identify, with cycle phase-specific insights, pathways and cells associated with endometriosis. A zoom of how xCell signatures were evaluated and selected to be representative of endometrial tissue. Left: Permutation test: Microarray transcriptome data were permuted at the gene level to construct tissue-specific null distributions for xCell’s outputs of its 64 signatures. Right: Microarray transcriptome data from sorted cells were summarized and combined at different ratios into artificial mixtures. Then xCell was run on these mixtures. Middle: For each of the 64 xCell signatures, a two-score schematic was designed to evaluate its relationship with respect to each endometrial cell type identified in the single-cell RNAseq dataset (27). A specificity score (ratioNext) was agnostically quantified and each xCell signature was categorized as either targeted or nontargeted (NA: no ref) based on whether there is an endometrial cell type or subtype that the signature is targeting, and “On Target” or “Off Target” based on whether the top-ranking endometrial cell type is the signature’s intended target or not, respectively. **(B)** Principal component analysis (PCA) of samples, after batch correction, based on (left) transcriptome data or (right) cell-type enrichment scores and colored by menstrual cycle phase or disease stage.

Microarray data were obtained from a public dataset (GSE51981), which was first processed and batch-corrected, followed by differential expression and pathway enrichment analyses to ensure agreement of data processing with previous literature (**Figure 1**). **Table 1** describes the study population consisting of 105 samples across various disease stages (34 control, 24 stage I–II, 47 stage III–IV) and cycle phases (47 proliferative endometrium (PE), 24 early secretory endometrium (ESE), 34 mid-secretory endometrium (MSE)).

**Table 1:** Cohort Statistics.

Cycle-Phase	Disease severity			Lab origin			Total
	Control	Stages I–II	Stages III–IV	Giudice	Burney	Lessey	
PE	20	10	17	31	13	3	47
ESE	6	6	12	14	10	0	24
MSE	8	8	18	19	11	4	34
Total	34	24	47	64	34	7	105

Batch correction successfully mitigated laboratory-associated variations (**Figure S1**). Based on PCA dimensionality reduction plots, samples tended to cluster by disease/stage (case) versus control and by phase (**Figure 1B**). Heatmap clustering, focusing on genes highlighted as differentially expressed (FDR < 0.05, and log2 fold change >1) in any sample stratification, revealed strong clustering based on case versus control status (**Figure S2A**). Phase-stratified analysis revealed overall concordance in the results (**Figures S2B**–**D**). Several differentially expressed genes were identified across multiple phases, but also some phase-specific associations with endometriosis (**Figures S2B, C**). Among significantly upregulated genes, 79 were common to all menstrual phases such as *FOSB*, *FOS*, *JUNB*, and *EGR1*, and 182 were shared across at least two phases. In addition, there were 27 genes specific to ESE, 106 to MSE, and 428 to PE. Among the significantly downregulated genes, 246 genes were common across all menstrual phases, including *CTSZ*, *SNTN*, *AGR3*, and *OLFM4*, and 693 genes were shared across at least two phases. In addition, there were 64 genes specific to ESE, 201 specific to MSE, and 962 specific to PE. Stratified analysis allowed the identification of phase and disease stage where dysregulation occurred (**Supplementary Table S1**). For example, CTSW, with functional roles in natural killer cells and cytotoxic T-cells and identified as upregulated in disease in previous unstratified analysis (4), was upregulated only in MSE in our analysis. The endothelial cell-related gene HSP90B1 was only elevated in PE, MSE, and stage I–II endometriosis. Immune-associated genes GNLY and C1QA were upregulated only in stage I–II and MSE, respectively. Discordance across phases is consistent with previous reports (7, 28); however, it is important to note that arbitrary differentially expressed genes (DEG) cutoffs may have amplified some of these differences as fold–fold plots revealed a high degree of correlation between phase-stratified disease versus control DEG results (**Figure S2D**). Similarly, concordant DEG signatures were also observed when data were stratified by disease stage (**Figure S3**).

Pathway analysis, conducted with GSEA targeting MSigDB’s Hallmark Pathways, confirmed the general concordance noted with disease versus control DEG fold changes across stratifications and also recapitulated known biology in that many immune pathways were among those associated with disease. Interferon alpha and gamma responses, TGF-beta and IL-2 STAT5 signaling, and complement pathways were upregulated in endometriosis consistently across most menstrual phase and disease stage stratifications (**Figure S4**; FDR < 0.05). TNF alpha signaling and allograft rejection, on the contrary, were significantly downregulated in disease across all cycle phases (FDR < 0.05).

### 2.2 Evaluation of Applicability 64 Cell-Type Deconvolution Signatures to Endometrial Tissue

Cell-type deconvolution provides a powerful opportunity to computationally disentangle bulk transcriptomic data into individual cell types. After confirming the agreement between our data processing with previous literature, we turned toward applying this technique by adapting a gene set enrichment-based deconvolution method (26) for use in the human endometrium (**Figure 1**). The original method provided a comprehensive signature compendium for 64 classical human cell types derived from multiple organ types based on 6 human tissue consortia. To allow for discovery of new cell types and relationships between cell types, rather than using prior knowledge to select a subset of the signatures for analysis, we opted to utilize all signatures and rely on statistical metrics to infer likelihood that a cell type, of the 64, may be present in the endometrium.

As a summary, we first evaluated the statistical significance of xCell results using a permutation test. We then evaluated the specificity of xCell’s signatures toward endometrial cell types using a published scRNAseq dataset on healthy human endometrium. For in-depth evaluation on xCell’s signatures for immune subtypes, we performed immune cell only scRNAseq analysis on the scRNAseq dataset and examined the relationship between xCell’s immune subtype signatures and identified endometrial immune subtypes. Lastly we applied xCell enrichment analysis to artificial mixtures constructed from sorted endometrial cells to further validate our approach.

#### 2.2.1 Evaluation of Statistical Significance of xCell Output Using Permutation Analysis

**Figure 2:**
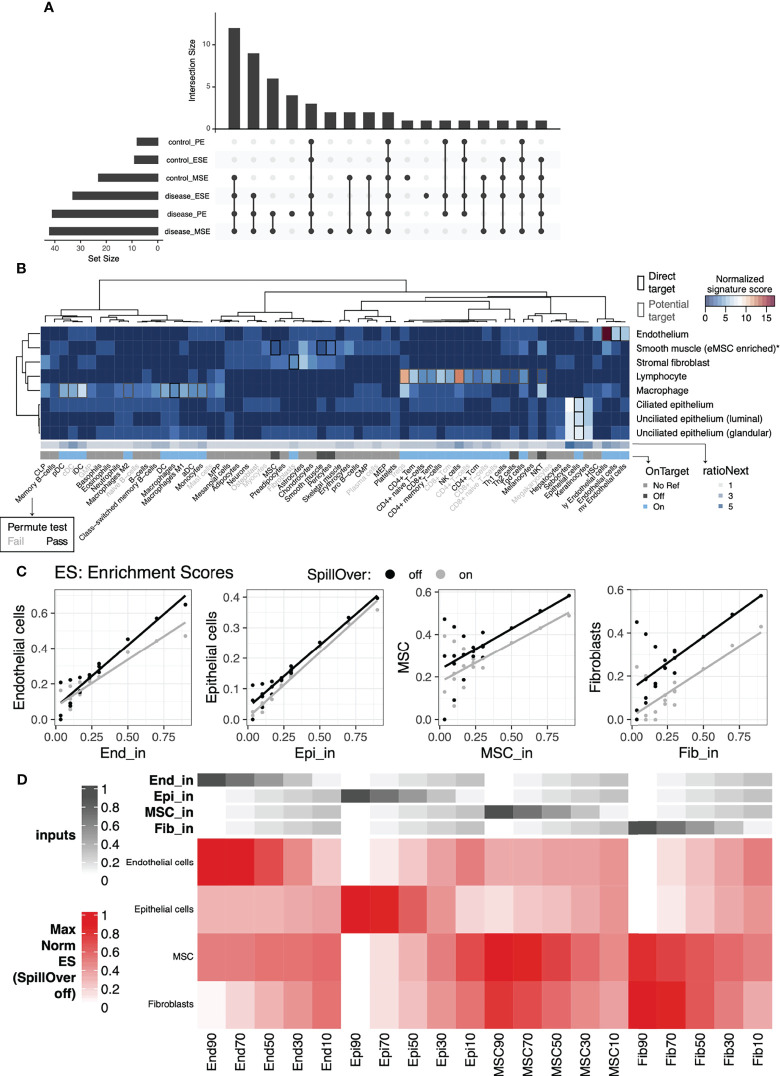
Cell-type specific signature validation for endometrial tissue. Evaluation of xCell’s 64-cell-type signature compendium and endometrial cell types identified *via* scRNAseq and artificial mixtures analysis from sorted cell types. **(A)** Upset plot showing patterns of conditions in which cell-type signature scores were significantly higher than permuted null distributions (top), as well as the sizes of each individual set (left). **(B)** Sensitivity (normalized signature score), specificity (RatioNext), and relationship (onTarget) between xCell’s 64 signatures with endometrial cell types identified at the single cell level. Shown in the heatmap are signature scores evaluated as percentage of genes in a given xCell signature that were differentially expressed between cells in an endometrial cell type compared to the remaining cells. Scores were normalized by row mediums. **(C)** Scatter plots of xCell enrichment scores (y-axis) versus input percentage (x-axis), for each input cell type, with least-squares regression line overlaid, for artificial mixtures with (gray) and without (black) the SpillOver step. **(D)** Heatmap of relative, normalized to the max across all mixes, enrichment scores with annotations at the top indicating the input percentage of each cell type. End, endothelial cells; Epi, epithelial cells; MSC, mesenchymal stem cells; Fib, eSFs.

xCell produces nonzero abundance scores for all cell-type signatures assessed, regardless of whether those cell types truly exist in the tissue. To overcome this, we estimated the statistical significance of enrichment scores using a permutation test (**Figure 1A**, right). Specifically, we permuted the gene labels of the bulk data of our tissue of interest and recalculated xCell enrichment scores 1,000 times to generate a dataset-specific null distribution of enrichment scores for each xCell signature. Statistical significance of scores from unpermuted data was then calculated relative to the null distribution to determine signatures for which enrichment scores were statistically significant, i.e., putatively above background. The analysis was performed separately for disease and control samples and for each cycle phase to ensure retention of cell types that might be abundantly present only in one condition or during a specific cycle phase (**Figures S5**, **S6**). xCell signatures passing the permutation test (ecdf_null_(median xCell score) > 90%) in at least one phase of one tissue condition were retained for further analysis and interpretation. Specifically, 50 of 64 xCell signatures passed and were deemed statistically significantly above background (**Figure 2A**). More signatures were deemed significant in secretory phases and in the disease condition (**Figure 2A** and **Figures S5**, **S6**). In total, 22 signatures were deemed statistically significant only in disease, and only platelets were deemed above background solely in control samples. Such a finding is in line with the presumed endometrial infiltration of additional immune cell types among women with endometriosis (29) and during secretory phases.

#### 2.2.2 Evaluation of the Specificity of Cell-Type Deconvolution Signatures to Human Endometrial Cells Using Single-Cell RNAseq Data

Even when the output of an xCell signature passes the permutation test, the associated abundance score does not necessarily reflect the behavior of its nominal target in the tissue of interest. Potential for inter-tissue transcriptomic difference of a cell type or ambiguity in naming a cell type can lead to low specificity of a signature to its nominal target. To overcome this challenge, we built two scores to evaluate xCell signatures’ specificity to (ratioNext) and relationship with (onTarget) known endometrial cell types (**Figure 2B**, see *Materials and Methods*) using a published scRNAseq dataset of the human endometrium from women without endometriosis (27). We plotted these two scores alongside all of our xCell outputs to help interpret the results in the context of endometrial cell types.

For xCell signatures whose direct nominal target cell type(s) were identified in the single cell dataset (**Figure 2B**, black boxes), we observed high-to-moderate specificity scores with “On target” classification for all except those targeting the cell type enriched with endometrial mesenchymal stem cells (low ratioNext, “Off target”) (30), which we refer to, herein, as eMSCs. Specifically, we found that all candidate eMSC-targeting signatures (MSC, pericytes, and smooth muscle cells) were more differentially upregulated in eSF than in eMSC. The results above were also validated by artificial mixtures constructed with purified endometrial cells of varying abundances, described below (**Figures 2C, D**).

For xCell signatures without a direct nominal target cell type in endometrial tissue (onTarget=No Ref), we observed high-to-moderate specificity scores for keratinocytes, sebocytes, skeletal muscle, and hematopoietic stem cell (HSC) signatures. Given the specialized biological function of keratinocytes, sebocytes, and skeletal muscle, it is unlikely that these cell types are present in the endometrial tissue. Their high ratioNext scores likely reflect the transcriptomic similarity between these cell types and endometrial cell types where they show the highest specificity (e.g., endometrial epithelial cells for sebocytes and keratinocytes). On the other hand, transcriptomic similarity between HSC and endometrial endothelial cells may suggest a relationship in developmental lineage.

Importantly, for many xCell signatures that do not have a direct nominal target cell type but can potentially target a subtype or a related cell type of an identified cell type (**Figure 2B** gray boxes in the heatmap), we observed a high-to-moderate specificity score and “On target” classification. Signatures that fall into this category consisted primarily of immune signatures, as well as signatures for microvascular (mv) and lymphatic (ly) endothelial cells. We reasoned that a high specificity score and a “Passed” permutation test suggest that the associated cell type/subtype likely exists in the single cell dataset but may have been concealed by more pronounced differences of major cell lineages when all cell types were included in the scRNAseq analyses. We therefore performed heterogeneity analysis on only immune cells in the endometrial dataset to explore endometrial immune cell heterogeneity and to aid further evaluation of xCell immune signatures.

#### 2.2.3 Annotation of Endometrial Immune Cell Types at Single-Cell Level Using xCell Signatures

**Figure 3:**
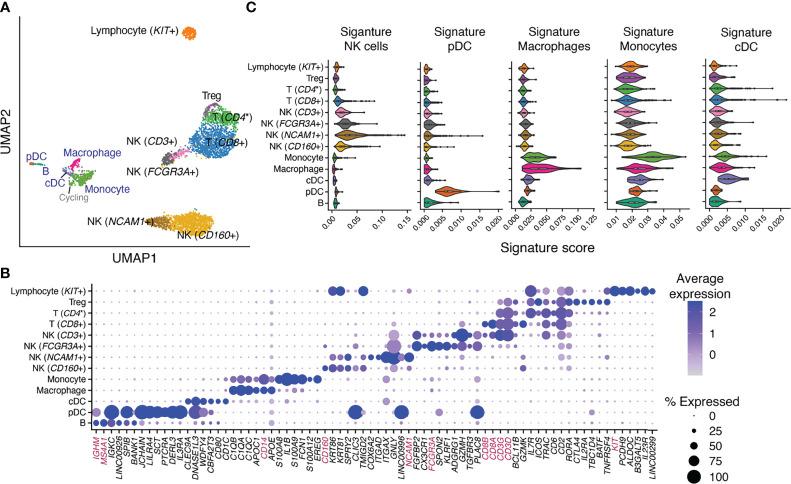
Identification and annotation of 13 immune cell types/subtypes in healthy human endometrium. **(A)** Dimensional reduction and cluster identification of endometrial immune cells from women with no gynecological conditions and in natural menstrual cycles. In blue are cell types/subtypes that were from the “Macrophage” cluster and in black were from the “Lymphocyte” cluster in the original analysis. **(B)** Top uniquely expressed genes for identified cell types/subtypes in **(A)**. In magenta are classical cell-type markers. **(C)** Score distribution of selected immune signatures compiled from 6 data sources in each identified immune cell type/subtype. NK, natural killer cells; pDC, plasmacytoid dendritic cells; cDC, classical dendritic cells; CD4*, *CD4* was uniquely but sparsely expressed in the cell subtype [(**Figure S8B**) and hence was not identified as a top uniquely expressed gene in **(B)**].

Immune-only heterogeneity analysis revealed 13 cell types/subtypes (**Figure 3A**): 5 were from the original “Macrophage” cluster and 8 were from the original “Lymphocyte” cluster (27). Classical immune cell-type markers allow broad annotation of these cell types/subtypes (**Figure 3B**). They alone, however, are not sufficient for confident cell-type annotation or for measuring similarity between identified cell types/subtypes and classically defined immune cell types for scenarios described below. We therefore iterated between a signature-based scoring method (31) using xCell’s signatures and classical marker expression to annotate the 13 identified cell types/subtypes. Most intriguingly, we identified one cell type that stemmed from the “Macrophage” cluster but expressed classical B cell receptor component genes (e.g., *JCHAIN*, *IGKC*) at a high level (**Figure 3B**). Our signature-based method revealed a distinct enrichment of plasmacytoid dendritic cell (pDC) (**Figure 3C**) and plasma cell signatures in the same cell type (**Figure S7C**). The pDC identity of this cell type was affirmed by the expression of genes uniquely identified in pDC (32) such as *CLIC3* and *SCT* (**Figure 3B**) and lack of expression of classical plasma cell markers such as *CD38* and *SDC1* (*CD138*) (**Figure S8A**). Similarly, we were able to discern among monocyte, macrophage, and classical dendritic cell (cDC) types, identify four NK cell subtypes (*NCAM1*+, *CD160*+, *CD3*+, *FCGR3A*+), one B cell type, and Tregs, whose annotation would not have been possible using classical markers alone due to marker co-expression in closely related cell groups. On the other hand, we identified one T cell subtype with high percentage of *CD8* expression [T (*CD8*+)] (**Figures 3B**, **S8B**) and another T cell subtype with sparse yet unique *CD4* expression [T (CD4*)] (**Figure S8B**). xCell’s signatures for CD4+ (**Figure S7B**) and CD8+ T (**Figure S7E**) cells, however, were not uniquely enriched in either of these T cell subtypes. Lastly, one lymphocyte cell type distinctly segregated from the rest of lymphocytes (**Figure 3A**) and uniquely expressed *KIT* and *IL23R* (**Figure 3B**). We, however, were not able to confidently annotate it using either the signature-based method or classical markers.

In summary, the aforementioned xCell signatures allowed confident annotation of immune cells in healthy endometrium. The unique upregulation of their scoring in endometrial immune cell types/subtypes further confirmed their applicability in deconvoluting the tissue. Moreover, xCell’s more than 40 immune cell signatures contain not only the aforementioned lineage-specifying signatures but also others that are either lineage- or function-specifying. We therefore scored all immune signatures in each of the 13 immune cell types/subtypes and plotted the result alongside deconvolution outcomes of each signature to guide interpretation (see below and **Figures 5**, **S7**, **S9**).

#### 2.2.4 Validation of the xCell Approach Using Artificial Mixtures From Sorted Cells

Finally, we validated that xCell enrichment analysis could be applied to microarray-based profiles of endometrial transcriptomes by applying the approach to artificial mixtures of microarray profiles of sorted cells. Microarray expression data from four FACS-purified endometrial cell types, endothelial cells (n=11 samples), epithelial cells (n=7), mesenchymal stem cells (n=28), and eSF (n=31) (9, 33–35), were median -summarized per gene, combined together into 20 different artificial mixtures, then analyzed with xCell (**Figures 2C, D**). The original method has a built-in compensation step to reduce spillover between closely related cell types. However, we observed 1) notable variations in deconvolution output in relation to which of the 64 signatures we selected as input, likely due to the use of a compensation matrix derived from *in silico* mixtures, and 2) signatures that appeared off-target in endometrial cells compared to their nominal cell type. We therefore disabled this step to ensure independence of outputs of 64 signatures. Confirming xCell’s utility, this analysis resulted in an overall positive correlation between the input ratio and output enrichment scores (**Figure 2C**). Yet also confirming the necessity of the aforementioned signature assessment metrics, it also revealed an interdependence of MSC and fibroblast signatures in that enrichment scores for these signatures appeared reliant on combined input amounts of both cell types, especially with low input abundance of eMSC and eSF (**Figure 2D**). We also confirm that removing the compensation step does not affect the overall trend in the deconvolution output for these signatures (**Figure 2C**).

### 2.3 Menstrual Cycle Phase and Endometriosis-Associated Changes in Cellular Composition of Human Endometrium

**Figure 4:**
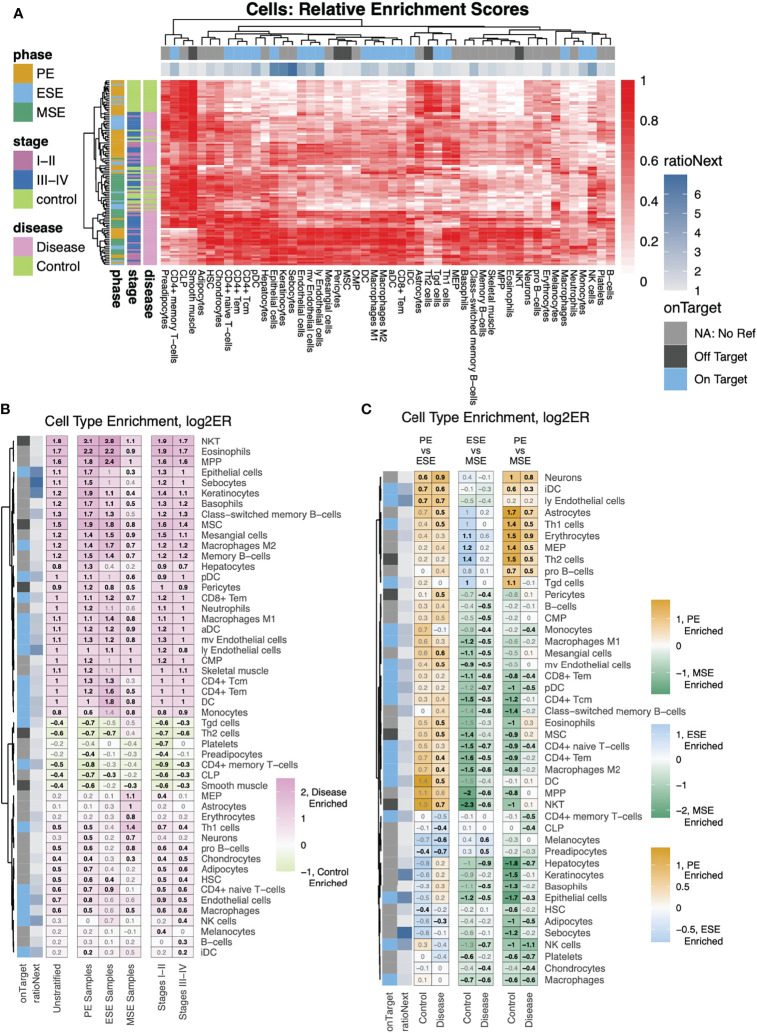
Differential analysis of cell-type enrichment. **(A)** Heatmap showing, for all samples, relative (compared to the max per cell type) enrichment scores, of all cells determined to be differentially enriched, for any stratification analyzed. **(B, C)** Cell-type enrichment analysis was performed based on FDR-corrected, two-sided, Mann–Whitney U tests between **(B)** disease versus control for either all samples (Unstratified) or stratifications to just specific phases (PE Samples, ESE Samples, MSE Samples) or between Stages I–II (labeled as such) or Stages III–IV (labeled as such) versus control among samples from all phases or **(C)** between phases among case and control samples separately. Shown are heatmaps of log2 fold changes for enrichment scores where only cell types with at least one significant comparison are shown. Numbers = log2FC with black color for statistically significant enrichments and gray color for nonstatistically significant enrichments.

With metrics built for evaluating the applicability of xCell’s 64 signatures to the human endometrium, we deconvoluted 105 human endometrium bulk transcriptomic profiles into the 64 cell types using the adapted xCell approach (**Figure 1A**). Signatures that passed the permutation test were retained for downstream analysis. The data were obtained in proliferative, early secretory, and mid-secretory phases of the menstrual cycle from women with stage I–II or more severe (stage III-IV) endometriosis, as well as those without disease (control) (**Table 1**). The overall clustering of the deconvoluted dataset was largely explained by disease versus control, followed by the menstrual cycle phase (**Figure 1B**, right). Cellular compositions that contributed to the changes in phase and disease were then assessed *via* stratified differential enrichment analysis (FDR < 0.05, no FC cutoff, **Figure 4**, **Table 2**) and interpreted alongside signature specificity metrics.

**Table 2:** Enrichment of cell types in each condition and menstrual phase and their fluctuation throughout the menstrual cycle.

Population (Signif. Stages Strat.When not both)	Disease vs Control		Variation across the cycle					
	Enriched in	Signif.	Control			Endo		
		Phase						
		Strat.	PE	ESE	MSE	PE	ESE	MSE
γδTcells	**Control (n = 34)**	**PE**	**+++** ^◊^	**++^~^**	**+** ^◊~^	++	++	++
CD4+ memory T cells		**PE**	+++	+++	+++	**+** ^◊^	**+++**	**++** ^◊^
Th2 cells		**PE, ESE**	**+++** ^◊^	**+++^~^**	**+** ^◊^ **^~^**	**++** ^◊^	**++**	**+** ^◊^
DC	**Endo (n = 71)**	**All**	+	+	++	**+++** ^◊^	**++** ^◊^	+++
iDC (stages III-IV)		**PE**	**+++** ^*◊^	**++** ^*^	**++** ^◊^	**+++** ^*◊^	**++** ^*^	**++** ^◊^
aDC		**All**	+	+	++	+++	++	+++
pDC		**PE, MSE**	**+** ^◊^	+	**++** ^◊^	**++** ^◊^	**++** ^~^	**+++** ^◊~^
Monocytes		**PE, MSE**	+	+	+	**++** ^◊^	**+** ^~^	**++** ^◊~^
Macrophages		**PE, MSE**	**+** ^◊^	**+** ^~^	**++** ^◊~^	**++** ^◊^	**++** ^~^	**+++** ^◊~^
Macrophages M1		**All**	+	**+** ^~^	**++** ^~^	+++	**++** ^~^	**+++** ^~^
Macrophages M2		**All**	**+** ^◊^	**+** ^~^	**++** ^◊~^	**+++** ^*^	**++** ^*~^	**+++** ^*~^
NK cells (Stages III-IV)		**None**	**+** ^◊^	**+**	**++** ^◊^	**+** ^◊^	**++** ^~^	**+++** ^◊~^
Neutrophils		**PE**	+	+	+	+	+	+
Eosinophils		**All**	**+** ^◊^	+	**++** ^◊^	**++** ^*^	**+** ^*^	++
Basophils		**PE, MSE**	**+** ^◊^	++	**+++** ^◊^	+++	**++** ^~^	**+++** ^~^
CD8+ Tem		**All**	**+** ^◊^	**+** ^~^	**++** ^◊~^	**++** ^◊^	**++** ^~^	**+++** ^◊~^
CD4+ naïve		**PE, ESE**	**+** ^◊^	**+** ^~^	**+++** ^◊~^	**++** ^◊^	**++** ^~^	**+++** ^◊~^
CD4+ Tcm		**PE, ESE**	**+** ^◊^	**+** ^~^	**+++** ^◊~^	++	**++** ^~^	**+++** ^~^
CD4+ Tem		**All**	**+** ^◊^	**+** ^~^	**++** ^◊~^	**+++** ^*^	**++** ^*~^	**+++** ^~^
Th1 (stages I-II)		**PE, MSE**	**+++** ^◊^	++	**+** ^◊^	**+++** ^*◊^	**++** ^*^	**++** ^◊^
Endothelial cells		**PE**	++	++	++	+++	++	+++
mv Endothelial		**All**	+	**+** ^~^	**++** ^~^	**+++** ^*^	++^*^ ^~^	**+++** ^~^
ly Endothelial		**All**	**++** ^*^	**+** ^*^	+	**+++** ^*^	**++** ^*^	+++
Epithelial cells		**PE, MSE**	**+** ^◊^	++	**+++** ^◊^	**+** ^◊^	**++** ^~^	**+++** ^◊~^
Keratinocytes		**All**	**+** ^◊^	++	**+++** ^◊^	+++	++	+++
Skeletal muscle cells		**PE, MSE**	+	+	+	++	++	++
Neurons (phase strats only)		**PE, MSE**	**+++** ^◊*^	**++** ^*^	**+** ^◊^	**++** ^*◊^	**+** ^*^	**+** ^◊^
Astrocytes (phase strats only)		**MSE**	**+++** ^◊^	++	**+** ^◊^	**+++** ^*◊^	**++** ^*^	**++** ^◊^
Erythrocytes (phase strats)		**MSE**	**+++** ^◊^	**++** ^~^	**+** ^◊~^	**++** ^◊^	++	**+** ^◊^
MPP		**All**	**+** ^◊^	**+** ^~^	**++** ^◊~^	++	**+** ^~^	**++** ^~^
HSC		**PE, ESE**	**+** ^*◊^	**++** ^*^	**+++** ^◊^	+++	+++	+++

Significant differences between: * = PE and ESE; ~ = ESE and MSE; ◊ = PE and MSE.

Abundance of the cell type = +/++/+++.

Unstrat., Unstratified.

#### 2.3.1 Deconvolution Results for Non-Immune xCell Signatures With Confirmed Specificity to Cell Types in Human Endometrium

**Figure 5:**
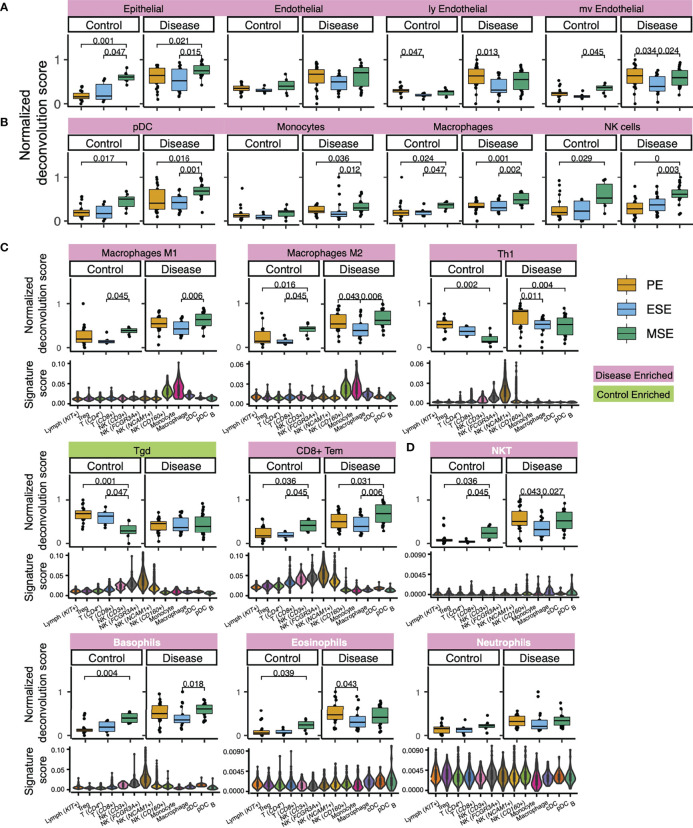
Deconvolution results and signature score distribution in single-cell data of select xCell signatures **(A)** with confirmed specificity to major endometrial non-immune cell types, **(B)** with confirmed specificity to endometrial immune cell types, **(C)** that are function specifying, and **(D)** lack representation in healthy endometrium but show endometriosis-associated statistically significant abundance increase. Significant p-values are marked on the figure. Signatures in panel **(D)** are colored in white. For violin plots in **(C, D)**, each violin represents an immune cell type/subtype identified *via* immune-only scRNAseq analysis on healthy endometrium (**Figure 3**). Signature score was calculated as the ratio between transcripts (UMI) that encode genes in the xCell signature to all transcripts (UMI) detected in each single cell (*Materials and Methods*).

Epithelial cells, eSF, and endothelial cells are the major non-immune cell types in the human endometrium. The specificity of their associated xCell signatures to the human endometrium was confirmed by our signature analysis (**Figure 2B**), with the exception for eSF whose signature failed the permutation test. For epithelial and endothelial cells, deconvolution analysis revealed both phase and endometriosis-associated changes. Epithelial cell enrichment scores in disease were notably elevated compared to control and also varied significantly across the menstrual cycle (**Figures 4B**, **5A**). Endothelial cells were enriched in disease in comparison to control (**Figure 4B**), with a slight increase in MSE versus ESE in control (**Figure 5A**). For both cell types, among all phases, a more significant rise in PE, compared to other phases, was observed in disease versus control (**Figures 4B**, **5A**). Disease-associated changes were also prominent in mv endothelial and ly endothelial signatures (**Figures 5A**, **4B**), both of which demonstrated high specificity to endometrial endothelial cells in our signature analysis (**Figure 2B**). While the ly endothelial signature had elevated PE scores compared to other phases in both disease and control, the PE-associated rise in the mv endothelial signature was higher in disease versus control (**Figure 4C**, **5A**).

#### 2.3.2 Deconvolution Results for Immune xCell Signatures With Confirmed Specificity to Cell Types in Human Endometrium

pDC, monocytes, macrophages, and NK cells were identified in our heterogeneity analysis of single-cell data from healthy endometrium (**Figure 3**), with confirmed specificity of their associated xCell signatures (**Figure 3C**) for deconvolution analysis. pDC enrichment scores were higher in disease over control across all cycle phases, reaching the highest enrichment score and statistically significant difference versus control in MSE (**Figures 5B**, **4B, C**). Similar patterns were observed for macrophage and monocyte scores, both signatures being enriched in disease across the cycle (**Figures 5B**, **4B, C**). In control, monocyte and macrophage enrichment scores were elevated in MSE (**Figure 4B, C**), whereas a more statistically significant rise in MSE was observed in disease for both (**Figures 5B**, **4B, C**). In both control and disease, NK cell enrichment scores increased notably in MSE compared to preceding phases (**Figures 5B**, **4C**). NK scores showed a slight yet statistically significant increase in stage III–IV endometriosis compared to control (**Figures 4C**, **5B**).

#### 2.3.3 Deconvolution Results for Immune xCell Signatures With Functional Applicability to Cell Types in Human Endometrium

As mentioned earlier, xCell’s comprehensive immune signatures include those that are function-specifying. Our signature-based annotation of the single-cell dataset revealed that some of these signatures were uniquely enriched in cell types/subtypes that we annotated by lineage and classical markers in healthy endometrium. For example, both macrophage M1 and M2 signatures are uniquely enriched in monocytes and macrophages in healthy endometrium (**Figure 5C**). Deconvolution results revealed an across-cycle increase in disease versus control for both signatures (**Figure 4B**), with an elevation in MSE compared to ESE in both disease and control (**Figure 4B**) with higher statistical significance in disease (**Figure 5C**). Both xCell’s DC and activated DC (aDC) signatures were enriched in monocytes, macrophages, and cDC cells at a single cell level, whereas immature DC (iDC) signature was elevated additionally in pDC and B cells (**Figure S7A**). Deconvolution results revealed overall increases in DC and aDC in disease versus control, with a dip in score dynamics (significantly lower DC score in ESE vs. PE; lower aDC score in ESE vs. MSE although not statistically significant) observed in disease (**Figures 4B**, **S7A**). Enrichment scores for iDC were elevated in PE compared to ESE and MSE in both disease and control (**Figure S7A**).

For some signatures, the lineage identity of cell types/subtypes where the signatures were uniquely enriched differed from the lineage identity of the signature. For example, Th1 signature was uniquely enriched in *NCAM1*+ and *FCGR3A*+ NK cells in healthy endometrium, Tgd signature was elevated in all NK cell types, and CD8+ Tem signature was elevated in all NK cell types and CD8+ T cells (**Figure 5C**). Deconvolution results showed across-phase decreases in both Th1 and Tgd signatures in control but deviating behaviors in disease (**Figure 5C**). CD8+ Tem signature had higher scores across all phases in disease compared to control and increased in MSE compared to the preceding phases in both disease and control (**Figures 5C**, **4B, C**).

#### 2.3.4 Deconvolution Results for Immune xCell Signatures With Low Specificity to Cell Types in the Healthy Human Endometrium Dataset

Based on classical marker expression, we identified *CD4* expressing T cell, *CD8*+ T cell, Treg, and B cell in the healthy endometrial single-cell dataset. xCell’s CD4+ or CD8+ cell-type signatures that passed the permutation test did not show unique enrichment in the respective cell subtypes (**Figure S7B**). Most of xCell’s CD4+ T-cell signatures had overall elevation in lymphocytes, which explained the moderate ratioNext scores received in our signature analysis and suggests that their deconvolution outcome likely reflects the collective abundance of lymphocytes.

Although xCell’s Treg and cDC signatures show moderate enrichment in Treg and cDC identified in the single-cell dataset, deconvolution results of these signatures did not pass our permutation test (**Figures 2B**, **S5**, **S6**, **S7A, B**).

Intriguingly, xCell signatures for B-cell types generally scored higher in “Macrophage” cell types/subtypes than in “Lymphocyte” cell types/subtypes (**Figure S7C**). This may be due to lower numbers of B cells as well as the shared antigen-presenting functions between B cells and macrophages leading to joint clustering of these cell types. However, only naïve B-cell signatures showed moderate elevation specific to B cells identified in the single-cell dataset, yet this signature did not pass the permutation test. Enrichment scores for all B-cell types were low and relatively constant in control, except for class-switched B cells, which displayed a slight increase across cycle, and for pro B-cell scores, which were elevated in PE and ESE. In disease, all B-cell scores were elevated in all phases compared to control, although with a higher extent in PE compared to other phases (**Figures 4B, C**, **S7C**).

#### 2.3.5 Deconvolution Results for xCell Signatures Lacking Representation in the Healthy Human Endometrium Dataset (Enriched in Disease)

For several xCell signatures that passed the permutation test, there were no associated cell types in the healthy single cell dataset. Thus, we have less certainty about how applicable these signatures are for potential endometrial versions of their cognate cell types. These signatures include NKT cells, neutrophils, eosinophils, basophils (**Figure 5D**), common myeloid progenitors (CMPs), and multipotent progenitors (MPPs) (**Figure S7D**), which all have relatively low enrichment scores in control (**Figures S5**, **S6**) and consistently higher enrichment scores, across the cycle, in disease. The increase is at least twofold higher in PE for all of these cell types (**Figures 4B**, **5D**, **S7D**). For NKT, eosinophils, basophils, and MPPs, a statistically significant increase was also observed in control MSE compared to a proceeding phase, although xCell’s basophil signature was enriched in two NK subtypes (**Figure 5D**).

As with our specificity analysis, keratinocyte and sebocyte enrichment scores correlated closely with epithelial cell scores (**Figures 4A**, **S9**). HSC and endothelial signatures also correlated in the single-cell dataset (**Figure 2B**), although their enrichment score results deviated slightly in ESE (**Figures 4A**, **5A**, **S9**).

In both disease and control, we report a steady across-cycle decrease in erythrocyte, neuron (**Figure S9**), and megakaryocyte-erythroid progenitor (MEP) signature enrichment scores and an overall elevation in disease (**Figure S7D**). Increased enrichment scores were also observed for the common lymphoid progenitor (CLP) (**Figure S7D**) signature in control and mesangial cell signature in disease (**Figure S9**).

## 3 Discussion

In this work, we comprehensively examined the cellular composition of the human endometrium across the menstrual cycle in women with and without endometriosis, *via* integrated bulk tissue deconvolution and scRNAseq analysis. Our approach was uniquely designed such that we leveraged a large sample size of bulk data, a comprehensive signature compendium for 64 classical human cell types based on 6 human tissue consortia, a GSEA-based deconvolution method, and a high resolution of single-cell RNAseq data—mitigating limitations inherent in each factor. Importantly, while benefitting from the comprehensiveness of the 64 signatures, we designed statistical metrics to evaluate the applicability of each to the human endometrium to ensure statistical significance and guide interpretation. With this approach, we identified cell types with altered enrichment in one or multiple menstrual cycle phases of women with endometriosis versus controls without disease. Also, novel transcriptomic-level signatures for 13 immune cell types/subtypes in healthy endometrium, not heretofore reported in endometriosis, including pDC and monocytes, were identified. The positive enrichment of these transcriptomic signatures might indicate the presence in the endometrium of previously unidentified cell types, or even phenotypes among known cell types, that had not been previously investigated (see discussion below for NK and T-cell subtypes).

### 3.1 Contributions of Non-Immune Cells to Endometriosis

Our signature evaluation confirmed the specificity of xCell’s signatures to most major non-immune endometrial cell types, including epithelial cells and endothelial cells, but not fibroblasts. We observed increased enrichment scores in PE with endometriosis for epithelial cells and endothelial cells. This is consistent with observations of increased endothelial proliferation in women with endometriosis and menorrhagia versus controls (36–38).

eMSC is an endometrial cell type that exhibits mesenchymal stem cell characteristics *in vivo* (39) and *in vitro* (9, 30). Based on different characterization metrics, this cell type has been referred to as mesenchymal stem cells (9, 30, 40), pericytes (35), perivascular cells (41), or smooth muscle cells (27), each of which is represented by a different xCell signature. Our evaluation using both single-cell data and artificial mixtures discovered the lack of specificity of xCell’s signatures (MSC, pericyte, and smooth muscle cell) to eMSC, especially with low eMSC abundance (**Figure 2C**), due to concurrent expression of these signatures in eSF. This observation confirms the close relationship between these two endometrial cell types and their common association with progenitor MSC and pericytes. eMSCs are implicated in endometriosis (9, 30), and future studies should use unique markers identified for this cell type, such as *RGS5*, *GUCY1A2*, and *NOTCH3* (9, 27, 35).

xCell’s fibroblast signature did not pass the permutation test despite receiving a moderate ratioNext score and “onTarget” classification (**Figure 2B**). This discrepancy may be due to many factors. Firstly, expression levels of fibroblast signature genes that passed the thresholds for ratioNext calculations (i.e., adjusted p-value and log2(fold change), *Materials and Methods*) often showed only the borderline fold changes, and as Subramanian et al. explain, signatures of this nature can be expected to score poorly in a gene set enrichment-based method (42). Furthermore, seemingly unrelated signatures such as chondrocytes, astrocytes, smooth muscle, and MSC showed the highest enrichment for the eSF cluster of the single-cell data (**Figure 2B**), and artificial mixture analysis confirmed that one of these cognate cell types, eMSC, could contribute to fibroblast-signature enrichment scores (**Figures 2C,D**). Our signature evaluation method (**Figure 1A**) thus considered output of this signature with low confidence. It is known that eSF have their own unique phenotype that is distinct from other fibroblasts of the body in many ways, and that expression profile as well with hormonal changes in the endometrium (9, 27, 35). Future studies should use a published dataset (9, 27, 35) to identify signatures that are specific to human eSF.

### 3.2 Contributions of Immune Cells to Endometriosis

Our scRNAseq analysis identified 13 transcriptomically distinct immune types/subtypes in healthy endometrium, which were previously broadly categorized into lymphocytes and macrophages (27). The use of a signature-based method (31) and classical cell-type markers allowed us to confidently annotate pDC and monocytes, which have not been confidently identified at single-cell resolution or functionally examined in the endometrium, but have been characterized collectively with cDC1 by marker IRF-8 (43). With confirmed applicability of xCell’s signature for both cell types in the endometrium, our deconvolution results revealed relative increases in pDC and monocytes during MSE in women with endometriosis (**Figures 4**, **5**), suggesting likely involvement in inflammation. pDC have known involvement in the inflammatory response normally and in pathologic settings through interaction with vasculature and T cells (44). Increased monocytes are congruent with the increased expression of monocyte chemoattractant protein-1 (*MCP-1*) in the endometrium of women with endometriosis (45, 46). Moreover, we observed a greater increase in monocytes in women with stage III–IV endometriosis, which may contribute to a lower implantation rate and live birth rates compared to women with stage I–II disease (47).

We have previously shown that endometrial macrophages (M1 and M2) in endometriosis are predominantly pro-inflammatory (10). Phase- or disease-stratified abundance quantification of endometrial myeloid cell types, including macrophages, monocytes, and dendritic cells, however, is limited. Here we report the unique markers that discriminate diverse endometrial myeloid cell types/subtypes for future studies.

Our scRNAseq analysis of immune cells in healthy endometrium has identified immune cell types/subtypes that are beyond the definition of xCell’s 64 signatures, such as four NK cell subtypes, one CD8+ T-cell subtype, one CD4 expressing T-cell subtype, and a *KIT*+ lymphocyte cell type. Intriguingly, xCell’s Th1, Th2, Tgd, and CD8+ Tem signatures were more enriched in NK cell subtypes rather than the T-cell subtypes in the single-cell dataset (**Figures 5**, **S7**). Therefore, the xCell outputs of these signatures across the cycle and in endometriosis likely reflect the changes of endometrial NK cell abundance or phenotypes more than the changes in the nominal cell types. However, these results do not conclusively suggest that these cell types are not present in the endometrium with or without disease, especially considering Th1 involvement in cytokine secretion and Tgd intraepithelial presence. Rather they are likely low in abundance, and their transcriptomic signals may be interfered by those of the more abundant NK cells. Lastly, although Tregs and cDC were identified in the scRNAseq dataset of healthy endometrium and demonstrated moderate enrichment of associated xCell signatures (**Figures 3**, **S7A, B**), their xCell signatures did not pass our permutation test. Previous studies have identified both cell types in eutopic endometrium (43, 48–51) and have shown that Tregs increased in abundance during the secretory phase in women with endometriosis compared to controls in both the eutopic endometrium (52, 53) and peritoneal fluid (54) with potential interplay with eSF (55). Enriching for aforementioned cell types/subtypes with classical markers and single-cell level identification and analysis is warranted in future studies.

Notable for some xCell signatures that passed our permutation analysis are the elevated abundance scores of eosinophils, neutrophils, basophils, NKT, and immune progenitors in the endometrium of women with endometriosis and their absence in women without disease and in the annotations of the scRNAseq dataset of healthy endometrium. Eosinophils, initiators of inflammatory responses, were enriched in all phases of the cycle, compared to the control endometrium wherein they appear mainly during menses, confirmed herein and by others (56). Thus, eosinophils likely contribute to the pro-inflammatory phenotype observed in bulk-tissue analysis of the endometrium from women with endometriosis. Our finding of neutrophils, key participants in the innate immune response to foreign pathogens and enriched in the endometrium of women with endometriosis and independent of the cycle phase, compared to controls, is consistent with other reports, although others found cycle dependence of this cell population in women with versus without disease (57, 58). Basophils also initiate inflammatory responses and were found herein to be enriched in the endometrium of women with disease in PE and were significantly increased throughout the cycle. We are unaware of other reports on this cell type in the endometrium of women with endometriosis, and this finding warrants further study.

### 3.3 Comparison to Prior Work

Our results generally agree with a prior deconvolution study on the endometrium from women with and without endometriosis (59), although fewer xCell signatures with disease-associated changes (9 in total) were identified compared to our study. Differences may be due to our adapted usage of the deconvolution method. Additionally, this study (59) did not design or apply metrics for statistical significance evaluation and result interpretation or develop *de novo* identification of normal endometrial immune cell signatures to enrich the xCell data interpretation in the endometrial context.

### 3.4 Strengths and Limitations of This Study

There are several limitations in this study. One is the limited sample size, especially in the ESE phase. Another limitation of our current approach is its limited capacity in inferring cell-type-specific phenotypic state. Although such insights can still be inferred for phenotype-specifying signatures, such as several immune cell subtypes mentioned above, for signatures without tissue-matching phenotype specifications, such as fibroblasts, such insights cannot be obtained directly from the deconvolution results. This is remarkable as the eSF changes transcriptomically across the cycle and displays marked abnormalities in endometriosis (9, 60) and is a key regulator of successful embryo implantation. Other cell-type deconvolution tools such as Cibersortx (15) provide the possibility to infer cell-type-specific gene expression profiles through additive combinations of input cell-type signatures. Successful application of this approach requires that highly specific cell-type signatures be used and that all potential cell-type signatures be included (61).

Further studies leveraging single-cell technologies as well as integrating different types of omics measurements including proteomics, epigenetics, and others will enable further corroboration of our findings and linking transcriptional phenotypes with endometriosis-associated cell types, especially considering the decreasing cost of single-cell analysis through strategies such as multiplexing. Functional studies will help elucidate the roles these cell types play in disease.

Through integrated whole-tissue deconvolution and single-cell analysis, we identified endometrial cellular compositions that are dynamic across menstrual cycle phases and altered in women with endometriosis. Guided by our signature evaluation metrics, we report cell-type candidates—immune cell types/subtypes of myeloid lineage, as well as non-immune cells, including epithelial and endothelial cell types—that most likely contribute to the pro-inflammatory endometrial phenotype previously observed in women with endometriosis (4, 7). Our results can help guide the selection of cell types for functional evaluation of cellular mechanisms that contribute to or result from endometriosis. Moreover, our analytical framework can be used in studies of other tissue types.

## 4 Materials and Methods

An overview of all methods is shown in **Figure 1**.

### 4.1 Code and Experimental Data

#### 4.1.1 Whole Tissue Microarray

Microarray data for this study were obtained from GSE51981 (4), and all analysis was carried out in R. Sample metadata for disease severity were used to classify all samples into groups of stages I–II and stages III–IV, with ambiguously mapping samples (n=1) being subsequently removed in further analyses. Sample metadata for pathology were used to classify samples as endometriosis, no pathology [which included labels NUP (no uterine pathology) and NUPP (no uterine or pelvic pathology)], or “other,” with all “other” samples being left out, as such samples represent imperfect controls, for subsequent analyses. Additional samples were removed, which had ambiguous lab source annotation (n=1) or cycle-phase annotation outside of proliferative endometrium (PE), early secretory endometrium (ESE), or mid-secretory endometrium (MSE) (n=1). In the end, this led to a total of 105 samples, 71 from women with endometriosis and 34 from women with no uterine or pelvic pathology (controls) (**Table 1**).

#### 4.1.2 Sorted Cell Microarray

Microarray data from purified human endometrial cell populations (stromal fibroblasts, endothelial, epithelial, and mesenchymal stem cells) isolated by fluorescence-activated cell sorting (FACS) were from previous studies: GSE73622, GSE31152, GSE48301, and GSE97163 (9, 33–35). These were used in artificial mixes of pure cell types in determining the signature specificity of the xCell signatures (see below).

#### 4.1.3 Single-Cell Transcriptomics

Endometrial single-cell RNAseq data used to evaluate xCell signatures were collected as endometrial biopsies from women without endometriosis or uterine or pelvic pathology, as previously described (27) (GSE111976 and SRP135922). For this study, 10x data published in (27) were used. The definition of endometrial cell types and subtypes is described in Extended Data Figure S1 in (27). Annotations of each cell with regard to participant, cycle phase, and cell type or subtype are available in a Supplementary File “GSE111976_summary_10x_day_donor_ctype.csv.gz” under GSE111976.

#### 4.1.4 Code and Processed Data Availability

The code for reproducing these analyses is available on Github (https://github.com/dtm2451/EndometrialDeconvolution), and processed data are available on figshare (https://figshare.com/projects/Whole-tissue_deconvolution_and_scRNAseq_analysis_identify_altered_endometrial_cellular_compositions_and_functionality_associated_with_endometriosis/127208).

### 4.2 Microarray Normalization and Batch Correction

Background correction and quantile normalization were performed with the justRMA function of the affy package (62). Then batch correction was performed with ComBat (63) to reduce signals coming from the lab of origin, while protecting signals associated with the disease stage and the cycle phase. Direct principal component analysis (PCA) and the pvca package (64), which combines PCA with variance component analysis to estimate the proportion of variation in data that are associated with a set of potential sources, were used to assess the success of batch correction (**Figure S1**).

### 4.3 Differential Expression

Differential expression (DE) analysis was carried out *via* linear modeling using the Limma package (65) and the log-transformed and batch-corrected expression matrix as input. Simple, ~ single variable, formulas were used for linear model designs. When cutoffs for significant differential expression were used, they were FDR < 0.05, and abs log2 fold change > 1. Such analysis was run on various stratifications of the data (**Figures 1**, **S2**, **S3**). For unstratified (all samples) analysis, DE was run 1) between disease versus control, 2) between stages I–II or stages III–IV versus control, and 3–5) between all combinations of pairwise cycle phase comparisons (PE vs ESE, PE vs MSE, ESE vs MSE). DE was also run between stages I–II versus stages III–IV, but zero genes met DE cutoffs. For stage-stratified analysis, DE was run separately on control samples only, stages I–II samples only, and stages III–IV samples only between all combinations of pairwise cycle phase comparisons. Lastly, for phase-stratified analysis, DE was run separately for PE samples only, ESE samples only, or MSE samples only, 1) between disease versus control and 2) between stages I–II or stages III–IV versus control.

### 4.4 Gene Pathway Enrichment Analysis

Pathway enrichment analysis was performed on the Broad’s hallmark gene sets (obtained *via* https://www.gsea-msigdb.org/gsea/msigdb/collections.jsp#H) by gene set enrichment analysis. This was carried out with the fgsea function of the fgsea package (66) on log2 fold changes of all genes (both significant and nonsignificant), for all stratifications and differential expression comparisons, with additional parameters: minSize = 15, eps = 0, and maxSize = 1500. Pathways with FDR corrected p-values below 0.05 were considered differentially enriched (**Figure S4**).

### 4.5 Cell-Type Enrichment

Of the numerous deconvolution and enrichment methods, those that attempt to deconvolve a sample into additive mixtures of reference cell-type signatures have a strong reliance on both concordance between reference signatures and cell-type profiles of the target tissue, as well as on the presence of reference signatures for all cell types that might exist in the target sample. Given that we could not be certain that we would include signatures for all cell types that might exist in the endometrium, and that many immune cells profiled from the endometrium have shown noncanonical transcriptional profiles, we chose to use xCell’s enrichment-based approach, which is more robust to signature absence and inconsistencies, and includes signatures for 64 human immune and stromal cell types (including adaptive and innate immune cells, hematopoietic progenitors, epithelial cells, and extracellular matrix cells derived from thousands of expression profiles) (26). xCell was run on the log-transformed and batch-corrected expression data of human endometrial tissue described above. Due to uncertainty in the applicability of xCell signatures to endometrial tissue, only the rawEnrichmentAnalysis and transformScores steps were utilized for the calculation of enrichment scores. The spillOver adjustment step was not utilized due to notable deviations between xCell signatures versus nominal endometrial cell profiles, which would have been carried over into the compensation matrix derived from *in silico* mixtures of reference cells.

### 4.6 Filtration of Cell-Type Signatures Based on Permutation Analysis

As discussed by its authors, xCell often produces nonzero scores, which may result in false-positive interpretation for nonexistent cell types or unsuitable signatures. xCell signatures that might not apply well to endometrial samples were first identified based on comparison to a permuted background distribution (**Figures S5**, **S6**). A background distribution of enrichment scores was generated for every cell-type signature, and for each cycle phase, by running xCell with 1,000 permutations of our expression matrix where rownames (gene symbols) were shuffled. Significance testing was then performed for each cycle phase among control or disease samples individually. Median enrichment scores of all iterations, among the current stratification samples, formed the background distribution for each cell type. For a given stratification, a cell-type signature was then considered as expressed if the “true” median enrichment score, from the nonpermuted data, was greater than the 90th quantile of its background distribution. To ensure that cells present in only certain conditions might still be accurately assessed, this filtering procedure was run on a per-disease status and per-phase basis, and cell-type signatures were retained for future analyses as long as the median enrichment score was above the background cutoff for at least one stratification.

### 4.7 Evaluation of xCell Signatures Using Single-Cell Measurements of Endometrial Tissue From Women Without Endometriosis

Meanwhile, even when an xCell score is statistically significant, its contributing xCell cell-type signature may not be specific to its nominal cell-type target in the tissue of interest due to intertissue variability of the same cell type or ambiguity in cell-type naming.

To test sensitivity, for each of the 64 xCell signatures, a signature score was calculated with respect to each endometrial cell type identified in the scRNAseq dataset (27). To identify differentially expressed genes, Wilcoxon’s rank sum test (two-sided) was performed, and fold change (FC, dummy variable = 10^-2^) was calculated between cells from an endometrial cell type and the remaining cells. P-values obtained from Wilcoxon’s rank-sum test were adjusted for multiple comparison by the Benjamini– Hochberg’s procedure to obtain p.adj. A signature score was quantified as the percentage of genes in the given xCell signature that were differentially expressed between cells in an endometrial cell type compared to the remaining cells (p.adj < 0.05, log2(FC) > 1). For each of the xCell signatures, the resulting score was normalized by the median of scores of all eight endometrial cell types identified in the single-cell dataset (normalized s_i, j_ = s_i, j_/Med(s_i_,_1_, s_i_,_2_, … s_i_,_8_), where i is an xCell signature and j an endometrial cell type identified in the single-cell dataset).

Each xCell signature was categorized as either “reference” or “no-reference,” based on whether there is an endometrial cell type or subtype in the single-cell dataset that the signature is potentially targeting. A map between each xCell signature and each endometrial cell type was constructed to describe this relationship (**Figure 2B**, boxes). As shown in **Figure 1**, we kept this relationship relatively broad such that a signature is considered targeting a single cell type/subtype if it targets directly the identified endometrial cell type, or a subcategory of the identified cell type, or a related category of the identified cell type, to account for ambiguity in naming cell types and for potential existence of subtypes within the annotated cell populations.

Two specificity score metrics were then established. Given the target map, for the first specificity metric, “onTarget,” an xCell signature was tagged as “on-target” if the highest-ranking endometrial cell type from the single-cell expression data matches the cell type targeted by the xCell signature and “off-target” otherwise. Signatures without a clear reference cell type within the single-cell dataset were given an “NA” label (**Figure 2B**).

Separately, to evaluate how specific an xCell signature is to the endometrial cell type it represents, we calculated a “ratioNext” score representing the ratio between the highest and the second highest-ranking signature scores. Importantly, to avoid overpenalizing, if subtypes exist for the highest-ranking cell type (e.g., epithelial cells), scores in the subtypes were ignored in determining the second highest signature score (**Figure 2B**).

### 4.8 Identification and Annotation of 13 Immune Cell Types/Subtypes From Healthy Human Endometrium

Dimensional reduction was performed on cells from the two clusters annotated as “Lymphocytes” and “Macrophages” in the original analysis (27) using Seurat’s (v3.2.0) (67) implementation of uniform manifold approximation and projection (UMAP). Specifically, top 2,000 variable genes among the immune cells were identified *via* FindVariableFeatures(). Principal component analysis was performed *via* RunPCA() on the top variable genes. Dimension reduction was performed on the top 20 principal components (PCs) *via* RunUMAP() based on the distribution of variances explained by the top PCs. Cell types/subtypes were identified using Seurat’s FindNeighbors(dims = 1:20) and FindClusters(resolution = 0.6). For each identified cell type/subtype, FindNeighbors() and FindClusters() were iterated one additional round to identify further heterogeneity. A cluster is classified as a candidate immune cell type/subtype if it can be defined by statistically significant uniquely expressing markers. pDC and B cells were present in 4 samples, macrophages were present in 7 women, and the rest of identified immune types/subtypes were in all 10 women.

For each identified cell type/subtype, uniquely expressing genes were found *via* FindAllMarkers(only.pos = TRUE, min.pct = 0.25, logfc.threshold = 0.25, test.use = “wilcox”, slot = “data”) and ordered based on log2FC.

As elaborated in the text, annotation of each immune cell type/subtype was performed through iterative evaluation of classical marker expression, signature level scoring of xCell’s immune cell signatures, and RNA expression pattern of uniquely expressing genes identified above reported by the Human Protein Atlas (32). For signature scoring, we used the method reported in (31). Briefly, for each xCell’s immune signature, the score was quantified as the ratio between transcripts (UMI) that encode genes in the signature to all transcripts (UMI) detected in each single cell. We further examined the distribution of each signature in each identified immune cell type/subtype.

### 4.9 Validation of xCell Approach Using Artificial Mixtures From Sorted Cells

Microarray expression data from four cell types of FACS-purified endometrial cells (from participants with and without endometriosis) were used to generate 20 different artificial mixtures with varying proportions of each cell type. The microarray expression data from endothelial cells (n=11 samples), epithelial cells (n=7), mesenchymal stem cells (n=28), and stromal fibroblasts (n=31) were first summarized by their median expression for all probes. These median cell profiles were then additively combined into 20 different mixtures in which one cell type made up 10, 30, 50, 70, or 90% of the mixture, and the remaining cell types made up the remaining 90, 70, 50, 30, or 10%, respectively. xCell was then run on these mixtures both with and without the spillOver step (**Figures 2C, D**).

### 4.10 Differential Cell Type Enrichment Analysis

Log2 enrichment ratios (log2ER), between groups, were calculated for each cell type signature. P-values were generated by performing two-sided Mann–Whitney U tests between enrichment scores of all samples, between groups. These were then corrected for multiple hypothesis testing *via* the FDR method based on the number of signatures assessed. FDR < 0.05 was the sole cutoff used for differential cell-type enrichment. Such analysis was run on the same stratifications of the samples and for the same comparisons for each of those stratifications, as for differential gene expression analysis, described previously (**Figure 1**).

## Data Availability Statement

Publicly available datasets were analyzed in this study. These data can be found here: https://www.ncbi.nlm.nih.gov/geo/query/acc.cgi?acc=GSE51981, GEO, GSE51981.

https://www.ncbi.nlm.nih.gov/geo/query/acc.cgi?acc=GSE73622, GEO, GSE73622.

https://www.ncbi.nlm.nih.gov/geo/query/acc.cgi?acc=GSE31152, GEO, GSE31152.

https://www.ncbi.nlm.nih.gov/geo/query/acc.cgi?acc=GSE48301, GEO, GSE48301.

https://www.ncbi.nlm.nih.gov/geo/query/acc.cgi?acc=GSE97163, GEO, GSE97163.

https://www.ncbi.nlm.nih.gov/geo/query/acc.cgi?acc=GSE111976, GEO, GSE111976.

## Author Contributions

DB, WW, LG, and MS designed the study. DB, WW, and IB carried out computational data analysis. DB adapted xCell and performed deconvolution and bulk transcriptome analysis. WW designed the method for xCell’s signature evaluation and performed scRNAseq analysis. WW, DB, LG, MS, IB, JV-J, SH, IK, and JI contributed to data interpretation. SH, SS, JI, and KV organized the data and clinical annotations. All authors contributed to conceptualizing and editing the manuscript. All authors contributed to the article and approved the submitted version.

## Funding

The work has been supported in part by the National Institutes of Health, *The Eunice Kennedy Shriver *National Institute for Child Health and Human Development, and the National Centers for Translational Research in Reproduction and Infertility P50 HD055764.

## Conflict of Interest

The authors declare that the research was conducted in the absence of any commercial or financial relationships that could be construed as a potential conflict of interest.

## Publisher’s Note

All claims expressed in this article are solely those of the authors and do not necessarily represent those of their affiliated organizations, or those of the publisher, the editors and the reviewers. Any product that may be evaluated in this article, or claim that may be made by its manufacturer, is not guaranteed or endorsed by the publisher.
