# Supplementary material for: Whole-Tissue Deconvolution and scRNAseq Analysis Identify Altered Endometrial Cellular Compositions and Functionality Associated With Endometriosis
Source: Front Immunol. 2022 Jan 5;12:788315. doi: 10.3389/fimmu.2021.788315 (PMC8766492; doi:10.3389/fimmu.2021.788315)
Supplement: Supplementary file 1 [file DataSheet_1.pdf]

Supplemental Tables

Table S1: Differentially Expressed Genes, Case/Stage vs Control

Excel file with tabs representing: Unstratified DvC, PE sample DvC, ESE sample DvC, MSE sample DvC, Stages I-II vs control, Stages III-IV vs control

Table S2: Pathway Analysis, Case/Stage vs Control

Excel file with tabs representing: Unstratified DvC, PE sample DvC, ESE sample DvC, MSE sample DvC, Stages I-II vs control, Stages III-IV vs control

Table S3: Differentially Expressed Genes, Between Phases

Excel file with tabs representing: PE vs. ESE (Controls), PE vs. MSE (Controls), MSE vs. ESE (Controls), PE vs. ESE (Cases), PE vs. MSE (Cases), MSE vs. ESE (Cases)

Supplemental Figures

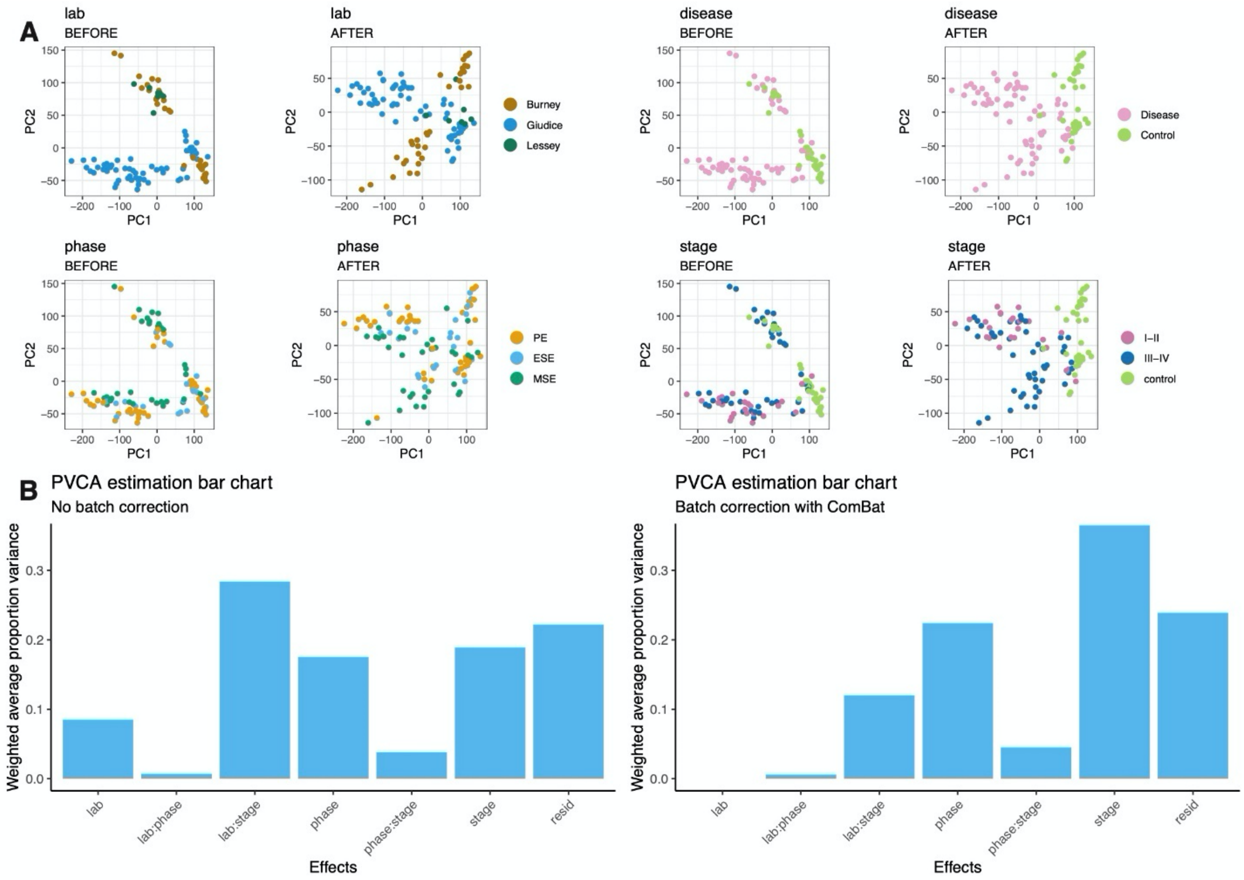

Figure S1: Batch correction reduces contributions from samples' lab origin

A. Principal component Analysis (PCA) of gene expression data (left) before and (right) after batch correction with ComBat with samples colored by different metadata. **B.** Bar plots showing the estimated variance associated with technical variables ("lab" of origin), biological variables (phase & stage), or interaction terms representing combinatorial contributions of these variables, (left) before and (right) after batch correction with ComBat, as estimated by pvca. Specifically, the pvca output values shown are weighted averages of the estimated variance contributions of each factor to every principal component (PC), weighted by the percent of total variance accounted for by each PC.

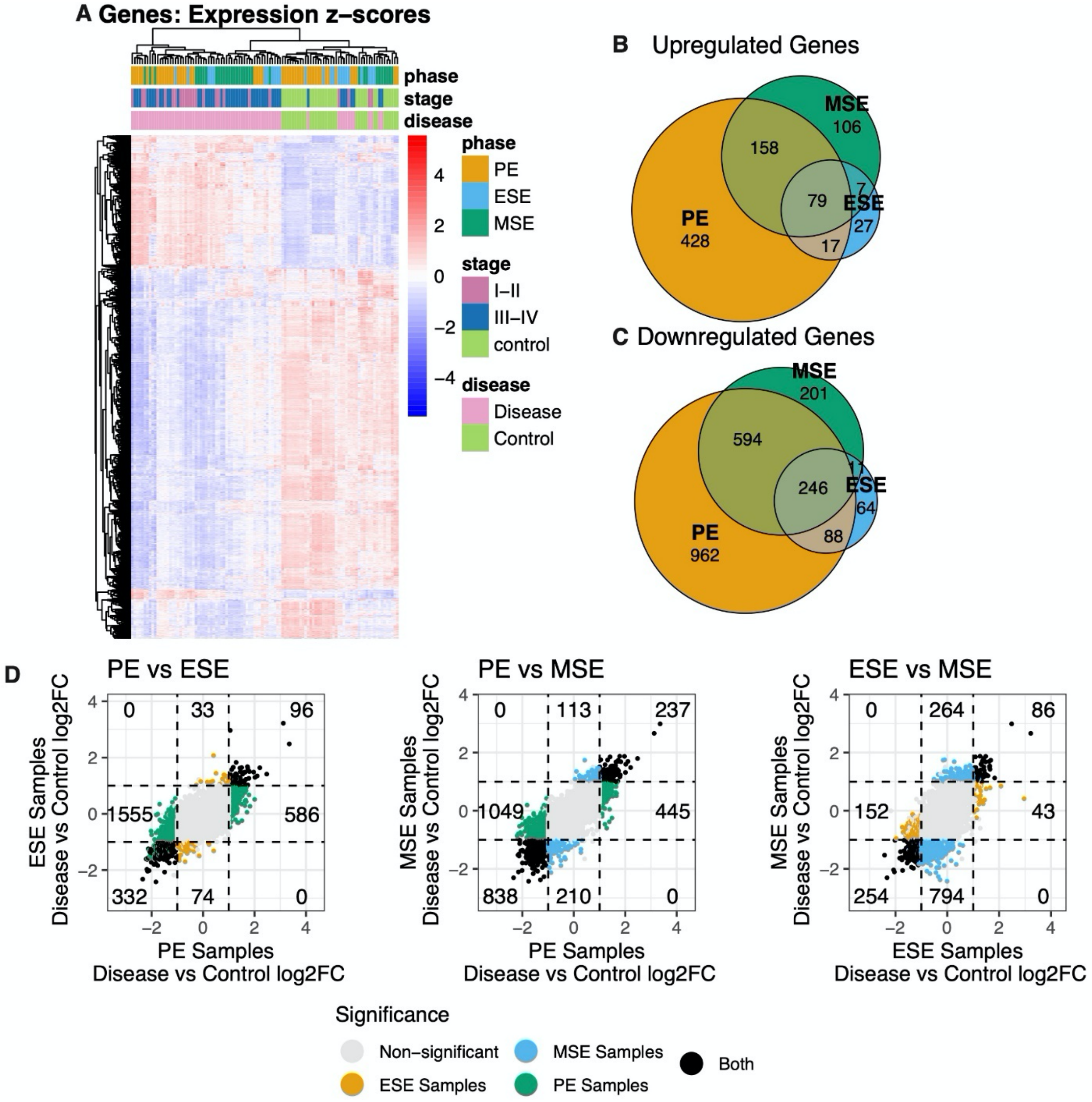

Figure S2. Disease versus Control Comparison on the Gene Level.

Differential gene expression was performed between disease versus control and Stages I-II or Stages III-IV versus control within various stratifications of the samples.

A. Heatmap showing, for all samples, (A) relative, z-score, expression of all genes determined to be differentially expressed**. B, C.** Venn diagrams comparing the composition of genes (B) up- or (C) down- regulated in disease versus control samples of each phase. **D.** Fold-fold plots comparing the log2 fold changes between disease versus control samples in the different phases.

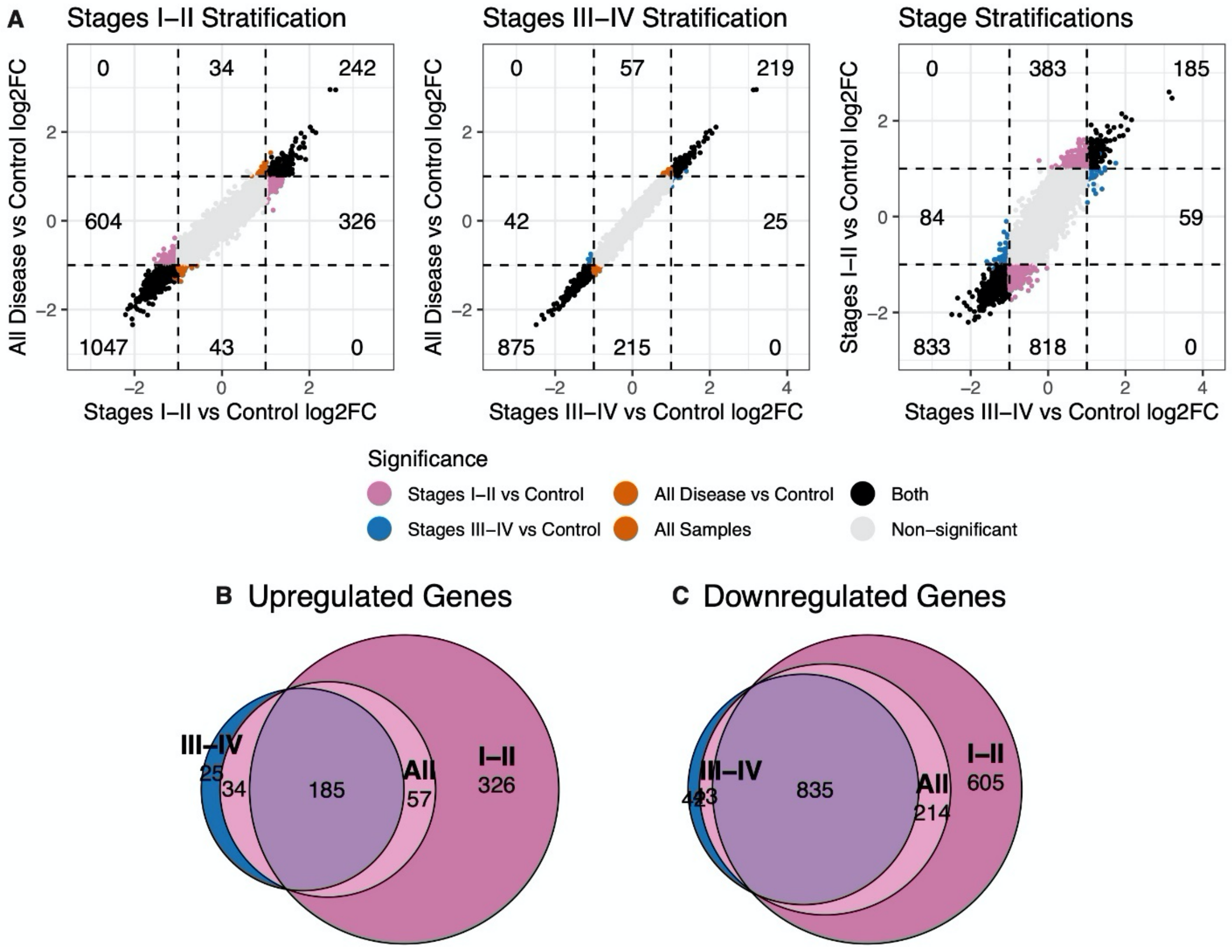

Figure S3: Disease and Stage versus control signature comparisons

Differential gene expression was performed between disease versus control and Stages I-II or Stages III-IV versus control samples. **A.** Fold-fold plots comparing the log2 fold changes between each of these comparisons. **B, C.** Venn diagrams comparing the composition of genes (B) up- or (C) down- regulated in disease/stage versus control samples.

Hallmark Pathway GSEA, NES

	Unstratified	PE Samples	ESE Samples	MSE Samples	Stages I–II	Stages III–IV
ADIPOGENESIS	2	2	2.4	2.2	1.9	2.1
ALLOGRAFT_REJECTION	−1.2	1	−1.2	−1.5	−1.1	−1.1
ANDROGEN_RESPONSE	2.2	2.4	1.8	2.5	2.3	2.2
ANGIOGENESIS	1.3	1.6	1.2	1.2	1.6	1.2
APICAL_JUNCTION	−1.5	−1.4	−0.9	−1.5	−1.5	−1.3
APOPTOSIS	1.4	1.5	1.7	1.5	1.4	1.4
BILE_ACID_METABOLISM	1.5	1.4	1.3	1.8	1.4	1.4
COMPLEMENT	1.4	1.5	1.5	1.3	1.4	1.3
DNA_REPAIR	1.9	1.8	2.2	1.6	1.7	2
E2F_TARGETS	3.2	3.2	3.2	2.1	3.1	3.2
EPITHELIAL_MESENCHYMAL_TRANSITION	1.4	1.7	1.1	−1.3	1.4	1.4
ESTROGEN_RESPONSE_LATE	1.2	1.2	1.5	1.2	1.1	1.3
FATTY_ACID_METABOLISM	2.1	2.1	2.1	2.4	2.1	2.1
G2M_CHECKPOINT	3	3	2.9	2.1	3	3
GLYCOLYSIS	1.7	1.8	1.8	1.8	1.7	1.8
HEME_METABOLISM	1.3	1.4	1.4	1.6	1.4	1.3
IL2_STAT5_SIGNALING	1.1	1.3	1.4	1.2	1.2	1.1
INTERFERON_ALPHA_RESPONSE	1.6	1.7	1.6	1.8	1.5	1.6
INTERFERON_GAMMA_RESPONSE	1.6	1.7	1.7	1.7	1.5	1.5
KRAS_SIGNALING_DN	−2.2	−2.4	−2.1	−2.1	−2.3	−2.2
KRAS_SIGNALING_UP	1.3	1.5	1.4	1.2	1.4	1.3
MITOTIC_SPINDLE	2.2	2.3	2.1	1.7	2.2	2.2
MTORC1_SIGNALING	2.6	2.7	2.4	2.4	2.6	2.6
MYC_TARGETS_V1	3.2	3.3	2.8	2.7	3.2	3.2
MYC_TARGETS_V2	1.5	1.3	1.7	1.2	1.3	1.7
MYOGENESIS	−2.2	−2.1	−1.7	−1.9	−2.1	−2.1
OXIDATIVE_PHOSPHORYLATION	2.6	2.5	2.7	2.7	2.4	2.7
P53_PATHWAY	−1	−1.2	1.3	−1.1	−1.3	−1
PEROXISOME	2	1.9	1.9	2.1	1.9	1.9
PROTEIN_SECRETION	2.8	3	2.4	3	3	2.8
SPERMATOGENESIS	1.4	1.4	1.4	1.2	1.4	1.4
TGF_BETA_SIGNALING	1.5	1.5	1.5	1.5	1.5	1.5
TNFA_SIGNALING_VIA_NFKB	−2.1	−1.6	−1.5	−2	−2	−2.1
UNFOLDED_PROTEIN_RESPONSE	2.1	2.2	1.8	1.9	2.1	2.1
UV_RESPONSE_DN	2.2	2.4	1.8	2.1	2.3	2.1
UV_RESPONSE_UP	−1.3	1.2	1.2	−1.3	−1.3	−1.4
XENOBIOTIC_METABOLISM	0.8	1	1.2	1.4	0.9	0.9

Figure S4. Pathway analysis was performed by GSEA using log2FC as input. Heatmap of GSEA normalized enrichment scores (NES) for hallmark pathways where only pathways with at least one significant enrichment are shown, and pathways related to the immune system are bolded. Case vs control comparisons stratified by phase and disease stage. NES with black color for statistically significant enrichments, and grey color for non-statistically significant enrichments.

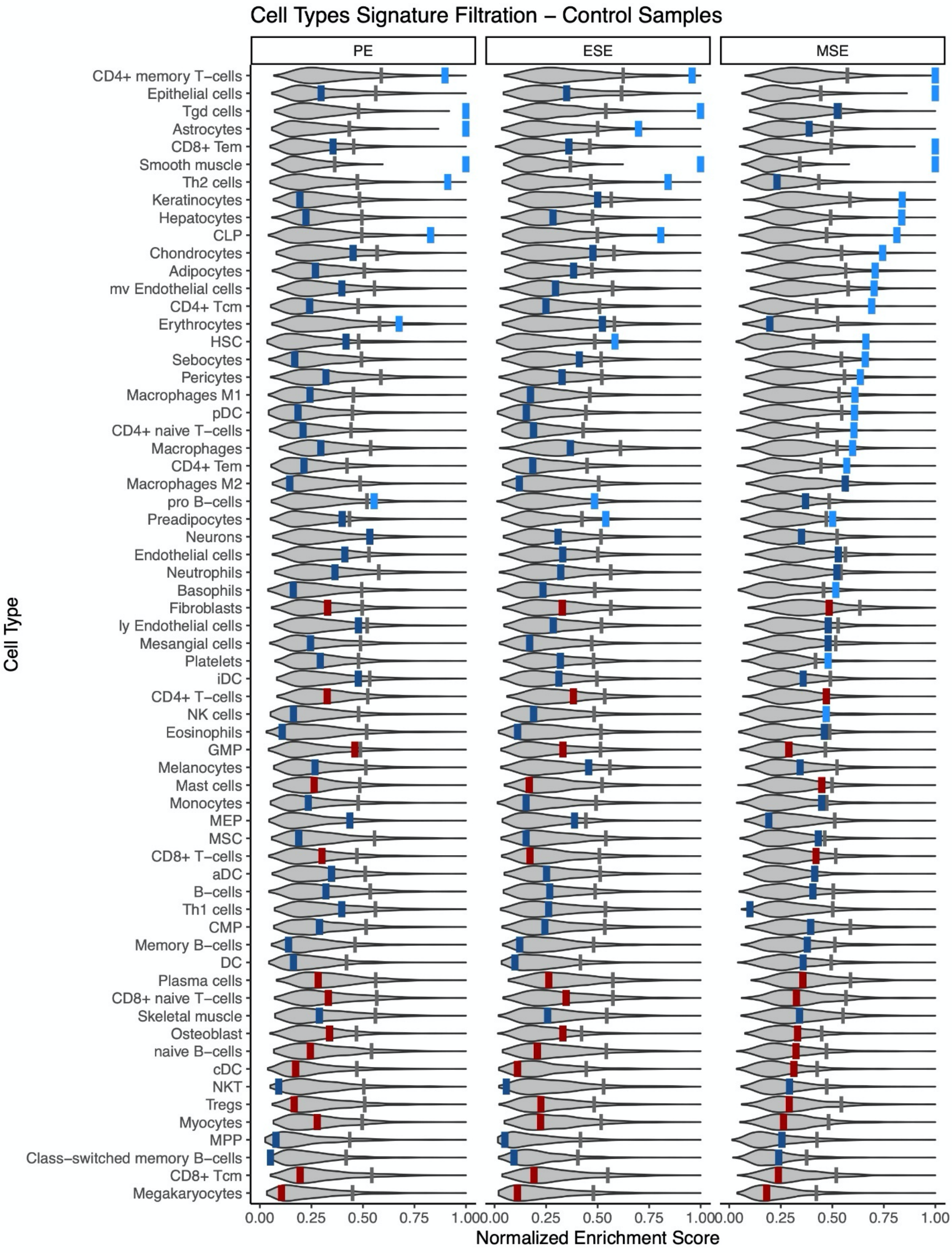

Figure S5: xCell Signature filtration based on a permuted background distribution (control samples)

Per-phase background distributions for cell type enrichment scores (ES) were generated by permuting gene symbols (row names) of the transcriptome matrix and then running xCell, 1000 times, then taking the median ES per phase of control and disease samples, in each iteration. Shown here are violin plots of these background distributions for each cell type signature, among control samples. Thin gray vertical lines represent the 90th quantile of these background distribution values. Thicker vertical lines represent the true median ES (from non-permuted data) for the given cell type with colors: Light blue = true median ES was greater than the background cutoff for this phase (signature retained); Dark blue = true median ES was less than the background cutoff for this phase, but not for another phase (signature retained); Dark red = true median ES was less than the background cutoff for all phases (signature filtered out for subsequent analyses)

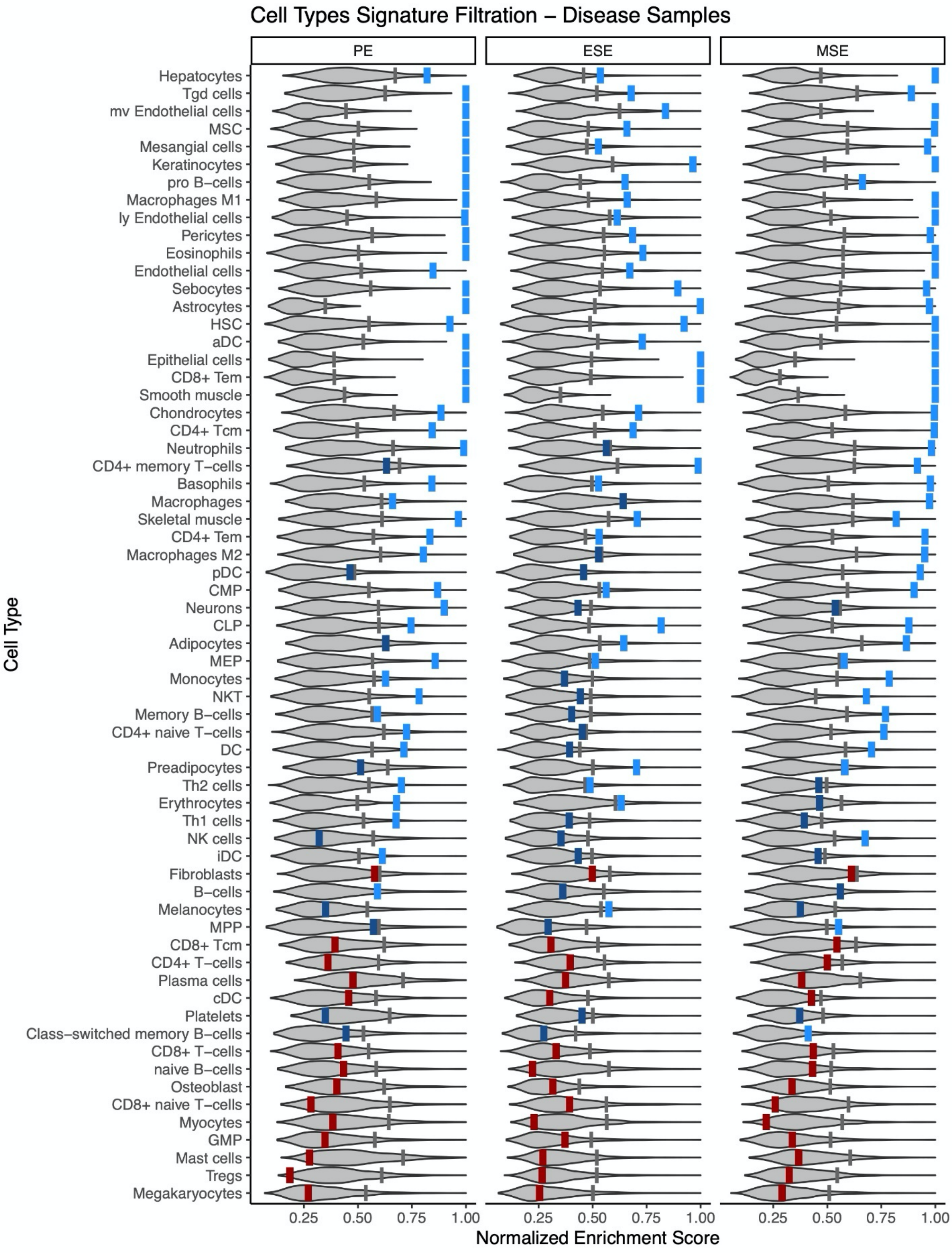

Figure S6: xCell Signature filtration based on a permuted background distribution (disease samples)

Per-phase background distributions for cell type enrichment scores (ES) were generated by permuting gene symbols (row names) of the transcriptome matrix and then running xCell, 1000 times, then taking the median ES per phase control and disease samples, in each iteration. Shown here are violin plots of these background distributions for each cell type signature, among samples from women with endometriosis. Thin gray vertical lines represent the 90th quantile of these background distribution values. Thicker vertical lines represent the true median ES (from non-permuted data) for the given cell type with colors: Light blue = true median ES was greater than the background cutoff for this phase (signature retained); Dark blue = true median ES was less than the background cutoff for this phase, but not for another phase (signature retained); Dark red = true median ES was less than the background cutoff for all phases (signature filtered out for subsequent analyses)

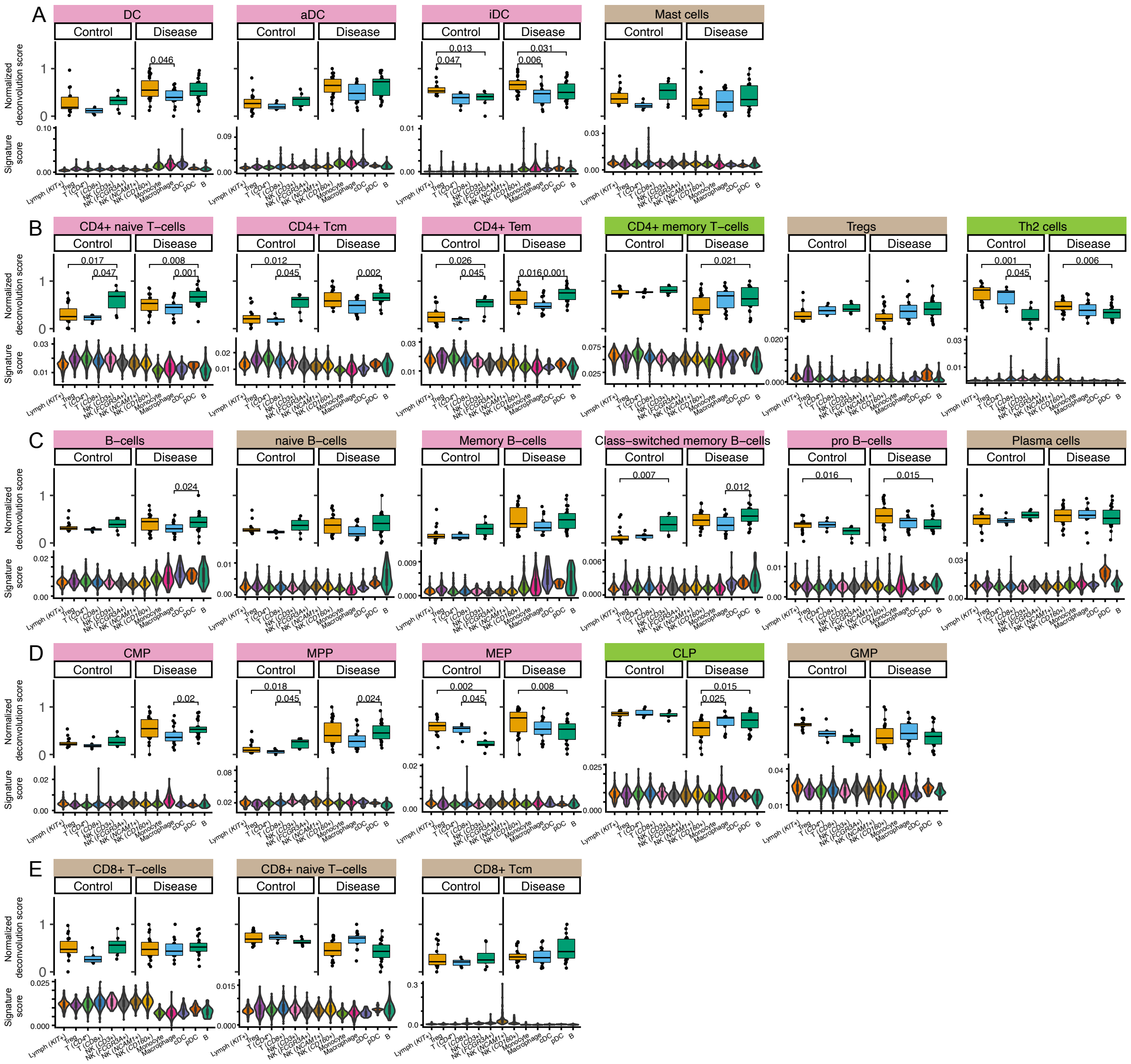

Figure S7. Deconvolution results and signature score distribution of xCell immune signatures of (**A**) myeloid lineage, (**B**) T cell types (**C**) B cell types (**D**) progenitors and (**E**) CD8+ T cell types. Signatures shown in **Figs. 3** and **5** are not repeated here. For violin plots in (C)(D), each violin represents an immune cell type/subtype identified via immune-only scRNAseq analysis on healthy endometrium (Fig.3A). Signature score was calculated as the ratio between transcripts (UMI) that encode genes in the xCell signature to all transcripts (UMI) detected in each single cell.

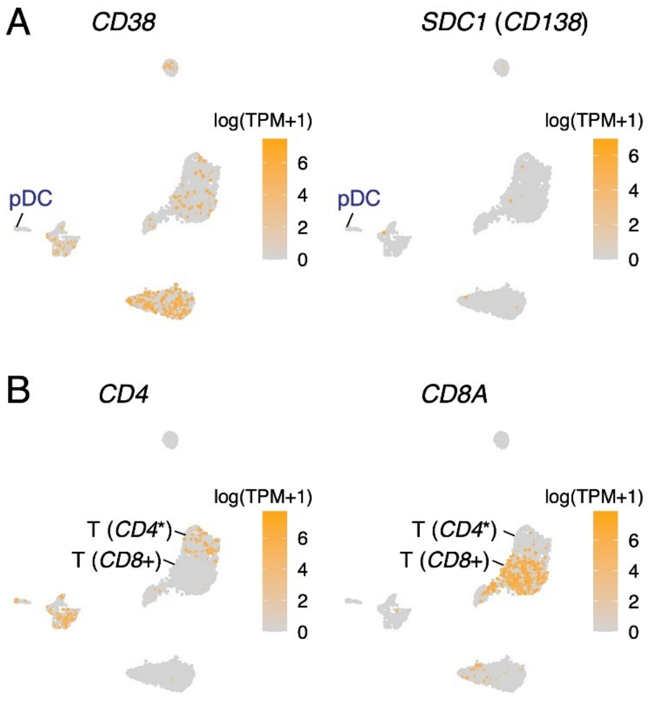

Figure S8. **Absence of expression of classical plasma cell markers in pDC (A) and expression of *CD4* and *CD8A* in CD4* T and *CD8*+ T cells, respectively (B)**, **identified in healthy human endometrium.** TPM: Transcript per million. Log is in natural log. *CD4**: *CD4* was uniquely but sparsely expressed in the cell subtype (**B**) and hence was not identified as a top uniquely expressed gene in (**Fig. 3B**).

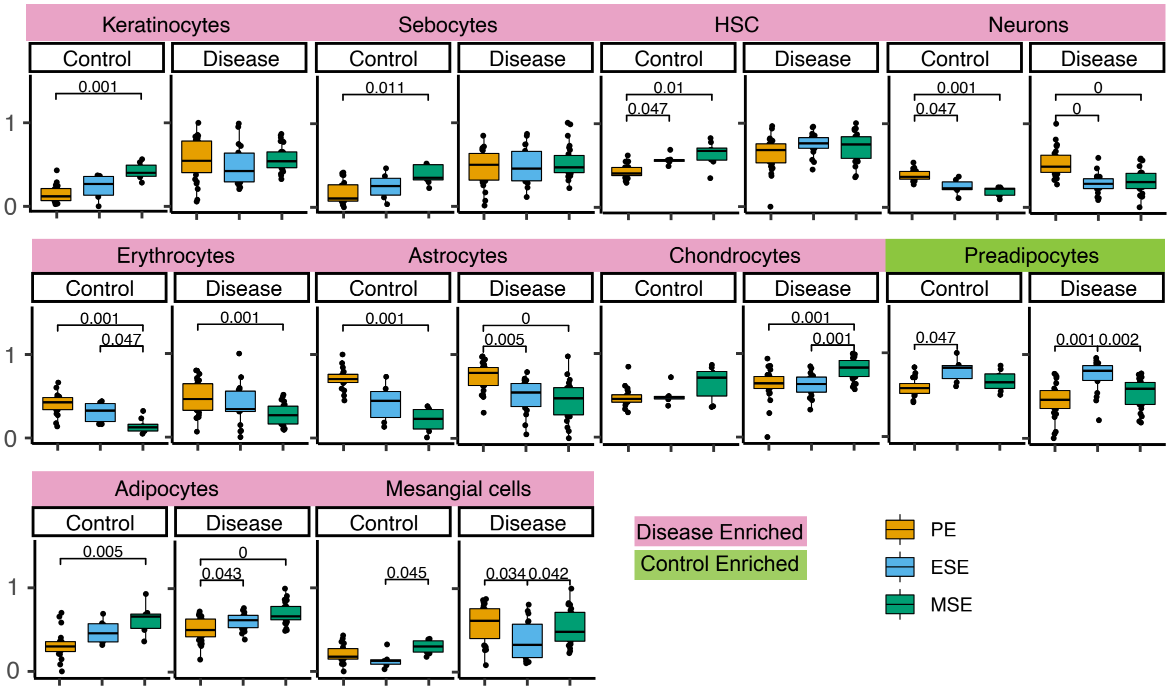

Figure S9. Deconvolution results of select xCell signatures. Enrichment scores are on the y-axis. Signatures shown in **Figs. 3** and **5** are not repeated here.
